# A retrospective analysis of oral and maxillofacial pathological lesions in a group of Egyptian children over 21 years

**DOI:** 10.1186/s12903-021-02037-6

**Published:** 2022-01-07

**Authors:** Mariam Mohsen Aly, Manar Abdul-Waniss Mohammed Abdul-Aziz, Marwa Aly Elchaghaby

**Affiliations:** 1 Pediatric Dentistry and Dental Public Health Department, Faculty of Dentistry, Cairo University, Giza, Egypt; 2 Oral and Maxillofacial Pathology Department, Faculty of Dentistry, Cairo University, Giza, Egypt

**Keywords:** Frequency, Oral maxillofacial, Pathological lesions, Children

## Abstract

**Objectives:**

The goal of the current study was to evaluate the relative frequency of oral and maxillofacial pathological lesions among Egyptian children.

**Materials and methods:**

Records of biopsies submitted to the department of oral and maxillofacial pathology from the year 1999 to 2019 were retrieved and reassessed for all cases under the age of 18 years. Information on age, sex, location of the lesion, and the histopathologic diagnosis was analyzed.

**Results:**

Over the course of twenty-one years, 1108 specimens were analyzed where reactive soft tissue lesions, which accounted for 397 (35.8%) of all cases ranked the highest presented category, followed by inflammatory odontogenic cysts, which accounted for 213 cases (19.2%). With 208 cases, the inflammatory radicular cyst was on the top of the most common 20 lesions, followed by pyogenic granuloma (160 cases). Malignancy was found in 19 cases, with soft tissue tumors (10 cases) being the most common, followed by salivary gland (5 cases) and bone pathologies (4 cases).

**Conclusions:**

The frequency of oral and maxillofacial pathological lesions among Egyptian children increased over the years but remained consistent with global trends.

**Clinical relevance:**

This is the first study evaluating the relative frequency of oral and maxillofacial pathological lesions among Egyptian children and provides an insight into the most commonly encountered pediatric pathologies. This may aid in the understanding of the most prevalent oral lesions that impact the pediatric population, as well as providing the key to early detection of lesions.

## Introduction

Pediatric oral and maxillofacial surgery is of particular interest in dentistry because the nature and severity of oral and maxillofacial diseases in children vary from those in adults. This is generally associated with their unique anatomical, and physiological considerations and the age-group predilection of some lesions. There can be marked changes in their histopathology, clinical behavior, and management since the nature of lesions changes in children as they grow and develop [1, 2].

Oral and perioral tissues can be affected by several diseases, including developmental, neoplastic, infectious, inflammatory, and reactive pathologies. The diagnosis of oral and maxillofacial pathologies can be challenging because of the diverse nature and the nonspecific features of diseases in this region [3].

The oral and maxillofacial area is subjected to a lot of harmful factors and carcinogenic chemicals that cause a diversity of diseases, some of which can be identified only on the basis of clinical signs and symptoms. For other soft tissue diseases and pathology with bony structures, an incisional or excisional biopsy is needed to confirm a definitive diagnosis [4].

The distribution of oral and maxillofacial lesions was assessed fundamentally to estimate their prevalence in the community, identify high-risk subpopulations, and optimize healthcare resources. Also, knowledge of the age, sex, and site predilections of different oral disorders are helpful in understanding their demographics [5].

Because of environmental differences, as well as the lifestyle of each group, the occurrence of these lesions varies significantly around the globe. Despite the World Health Organization (WHO) suggestions regarding the epidemiologic assessment of oral lesions, the majority of studies on oral conditions in children have been limited to the investigation of caries, periodontal disease, malocclusion, and dental trauma [6].

The majority of the previous epidemiological studies about the pattern and frequency of oral and maxillofacial pathology were limited to either particular age groups or specific categories of diseases. Studies about the relative frequency of oral and maxillofacial diseases from developing countries, particularly those in the Arab world are sparse [3, 7]

Despite the increasing literature about the incidence and prevalence of pediatric oral and maxillofacial pathology, there has been no research exploring the range and frequency in Egyptian children to the best of our knowledge.

## Materials and methods

### Study design and setting

The current study was a retrospective observational study undertaken at the Oral and Maxillofacial Pathology Department, Faculty of Dentistry, Cairo University. Ethical approval was obtained from the Ethics Committee of Scientific Research, Faculty of Dentistry—Cairo University with the approval number 19786. This study was recorded in clinicaltrials.gov with a registration number NCT04074395. The STROBE guidelines were used to confirm the reporting of the study.

### Participants

#### Inclusion criteria

- Children under the age of 18 years.
- Children with oral and maxillofacial pathological lesions who were biopsied at the Faculty of Dentistry, Cairo University.
- Inclusion of both soft and hard tissue pathologies

#### Exclusion criteria

- Records with missing original pathological slides.
- Incomplete data concerning age, sex, or histopathological diagnosis were excluded.

### Outcomes

This study aimed to assess:

- Primary outcome:
  - Frequency of oral and maxillofacial pathological lesions among Egyptian children.
- Secondary outcomes:
  - The number of cases in each diagnostic category and their percentages.
  - Age predilection for diagnostic categories.
  - Sex predilection for diagnostic categories.
  - Site distribution for diagnostic categories.

### Data sources and measurement

Biopsy records for children under the age of 18 years during the period from 1999 to 2019, were retrieved from the Oral and Maxillofacial Pathology Department, Faculty of Dentistry, Cairo University. Children diagnosed with oral and maxillofacial pathological lesions and biopsied at the Faculty of Dentistry, Cairo University with complete data were included in the study. The histopathological diagnosis of included samples was reassessed and confirmed through retrieval of original pathological slides.

The histopathological diagnoses were classified into 11 categories including periapical diseases, periodontal diseases, epithelial pathology, salivary gland pathology, odontogenic cysts, developmental non‐odontogenic cysts, benign odontogenic tumors, soft tissue tumors, bone pathology, dermatologic diseases, and allergies, and immunologic diseases. This classification system is consistent with the categories used by Jones and Franklin [8].

The study population was categorized into four groups 0–5 years, 6–10 years, 11–15 years, and 16–18 years. A databank, including the patient's age, sex, anatomical location of the biopsied lesion, histopathological diagnosis was developed using Microsoft Excel 2013.

### Statistical analysis

Data were shown as frequencies and percentages. Statistically significant differences were assessed using the Chisquare test where *P* < 0.05 indicated a statistically significant outcome.

## Results

During 21 year period (from 1999 to 2019), 6341 were presented for the histopathological report in the oral and maxillofacial department, Faculty of Dentistry, Cairo University. A total of 1108 specimens were received from children under the age of 18 years which correspond to 17.5% of the total number.

Out of the 1108 specimens, 412 (37.2%) cases were found in the age group of (11–15 years), 335 (30.2%) in the age group of (16–18 years), 280 (25.3%) in the age group of (6–10  years), and 81 (7.3%) in the age group of (0–5  years). There was an almost equal distribution of the lesions between males 549 (49.5%) and females 559 (50.5%).

The Site distribution analysis showed that the majority of the cases were located in the gingiva 354 (31.9%), the mandible 303 (27.3%), and the maxilla 262 (23.6%). While only 81 (7.3%) were located in the lip, 39 (3.5%) in the buccal mucosa, 31 (2.8%) in the palate, 23 (2.1%) in the tongue, and 15 (1.4%) in the floor of the mouth.

From the result of this study, there was an escalation in the prevalence of oral and maxillofacial lesions submitted for biopsy over the years in the Egyptian population. The specimens submitted for analysis were 114 (10.3%) in 1999–2003, 152 (13.7%) in 2004–2008, 293 (26.4%) in 2009–2013, and 549 (49.5%) in 2014–2019 in Table 1.

**Table 1:** Demographic data of the study sample

Demographic data	N	%
0–5 years	81	7.3
6–10 years	280	25.3
11–15 years	412	37.2
16–18 years	335	30.2
Female	559	50.5
Male	549	49.5
Gingiva	354	31.9
Mandible	303	27.3
Maxilla	262	23.6
Lip	81	7.3
Buccal mucosa	39	3.5
Palate	31	2.8
Tongue	23	2.1
Floor of the mouth	15	1.4
1999–2003	114	10.3
2004–2008	152	13.7
2009–2013	293	26.4
2014–2019	549	49.5

The diagnostic category with the highest presentation was the reactive soft tissue lesions 397 (35.8%) while benign soft tissue lesions represented only 35 (3.2%) of the lesions. The second most prevalent category was the inflammatory odontogenic cysts 213 (19.2%) followed by the developmental odontogenic cysts 93 (8.4%). On the other hand, the developmental Non-odontogenic cysts accounted only for 1% of the cases.

Reactive lesions were the most common type of salivary gland pathology, accounting for 61 (5.5%) of all specimens, while pleomorphic adenomas accounted for 5 (0.5%) of all pathologies.

Benign epithelial odontogenic tumors represented the highest prevalence 53 (4.8%) among odontogenic tumors compared to both mixed 25 (2.3%) and mesenchymal 19 (1.7%) tumors. Giant cell lesion was the most frequent bone pathological lesions 49 (4.4%) followed by fibro-osseous lesions 38 (3.4%) while benign bone pathology represented only 0.9% of the lesions.

Periapical lesions represented 47 (4.2%) of the total lesions where the majority of the cases were periapical granuloma 44 (4%). The least presented categories were allergies and immunologic disease 1 (0.1%), dermatologic diseases 2 (0.2%), and reactive epithelial pathological lesions 2 (0.2%) in Table 2.

**Table 2:** Distribution of study sample among categories with the most common lesion of each category

Categories	Subcategories	N	%	Most common lesion	N	%
Allergies and immunologic disease		1	0.1	Sarcoidosis	1	0.1
Bone pathology	Benign	10	0.9	Desmoplastic fibroma	3	0.3
	Giant cell lesion	49	4.4	Central giant cell lesions	41	3.7
	Malignant	4	0.4	Osteosarcoma	3	0.3
	Fibro osseous	38	3.4	Ossifying fibroma	27	2.4
Dermatologic diseases		2	0.2	White sponge nevus	1	0.1
				Lichen planus	1	0.1
Developmental Non-odontogenic cyst		11	1	Incisive canal cyst	6	0.5
Epithelial pathology	Benign	6	0.5	Squamous cell papilloma	4	0.4
	Reactive	2	0.2	Verruca vulgaris	1	0.1
				Traumatic ulcer	1	0.1
Odontogenic cysts	Developmental	93	8.4	Dentigerous cyst	38	3.4
				Odontogenic KeratoCyst	35	3.2
	Inflammatory	213	19.2	Inflammatory radicular cyst	208	18.8
Benign odontogenic tumor	Epithelial	53	4.8	Ameloblastoma	31	2.8
	Mesenchymal	19	1.7	Odontogenic myxoma	8	0.7
				Odontogenic fibroma	7	0.6
	Mixed	25	2.3	Odontoma	16	1.4
Periodontal diseases		22	2	Gingival fibromatosis	14	1.3
Periapical diseases		47	4.2	Periapical granuloma	44	4
Salivary gland pathology	Benign	5	0.5	Pleomorphic adenoma	5	0.5
	Malignant	5	0.5	Mucoepidermoid carcinoma	4	0.4
	Reactive	61	5.5	Mucous extravasation cyst (mcocele)	56	5.1
Soft tissue tumors	Benign	35	3.2	Hemangioma	9	0.8
	Malignant	10	0.9	Lymphoma	5	0.5
	Reactive	397	35.8	Pyogenic granuloma	160	14.4
				Peripheral giant cell granuloma	99	8.9
				Irritational fibroma	90	8.1
				Peripheral ossifying fibroma	38	3.4
Total number of lesions		1108	100		956	86.3

Regarding the most common lesions, inflammatory radicular cysts were ranked the most common lesion among the study sample with 208 cases followed by pyogenic granuloma with 160 cases and peripheral giant cell granuloma with 99 cases in Table 3.

**Table 3:** Distribution of the most common 20 lesions in the study

Most common 20 lesions	N	The percentage from the total population (%)
Inflammatory radicular cyst	208	18.8
Pyogenic granuloma	160	14.4
Peripheral giant cell granuloma	99	8.9
Irritational fibroma	90	8.1
Mucocele (mucous extravasation cyst)	56	5.1
Periapical granuloma	44	4.0
Central giant cell lesion	41	3.7
Dentigerous cyst	38	3.4
Peripheral ossifying fibroma	38	3.4
Ameloblastoma	31	2.8
Odontogenic keratocyst	30	2.7
Ossifying fibroma	27	2.4
Odontoma	16	1.4
Gingival fibromatosis	12	1.1
Gorlin's syndrome	12	1.1
Unicystic ameloblastoma	11	1.0
F. fibrous dysplasia	9	0.8
Hemangioma	9	0.8
Adenomatoid odontogenic tumor	7	0.6
Odontogenic fibroma	7	0.6

Regarding the comparison between the different age groups, a statistically significant difference was shown in the distribution of epithelial pathology, odontogenic cysts, benign odontogenic tumors, salivary gland pathology, and soft tissue tumors with *p* values 0.0431, 1.24E−05, 0.0001, 0.0001, and 3.48E−05 respectively in Table 4. While when comparing the prevalence of different pathological lesions between males and females, a statistically significant difference was found only in the occurrence of odontogenic cysts in favor of males with *p *values 0.0135, and salivary gland pathology in favor of females with *p* values 0.0028 in Table 5.

**Table 4:** Age distribution among study categories

Categories	Age distribution								*P* value
	0–5 years		6–10 years		11–15 years		16–18 years		
	N	%	N	%	N	%	N	%	
Allergies and immunologic disease	0	0.0	0	0.0	1	100.0	0	0.0	**0.6380**
Bone pathology	5	5.0	29	28.7	42	41.6	25	24.8	**0.3838**
Dermatologic diseases, immunologic	0	0.0	0	0.0	1	50.0	1	50.0	**0.8012**
Developmental, non-odontogenic cyst	2	18.2	3	27.3	5	45.5	1	9.1	**0.3004**
Epithelial pathology	0	0.0	5	62.5	0	0.0	3	37.5	**0.0431**
Odontogenic cysts	4	1.3	79	25.8	113	36.9	110	35.9	**1.24E−05**
Benign odontogenic tumor	5	5.2	7	7.2	45	46.4	40	41.2	**0.0001**
Periodontal diseases	2	9.1	6	27.3	9	40.9	5	22.7	**0.8884**
Periapical diseases	4	8.5	7	14.9	19	40.4	17	36.2	**0.4075**
Salivary gland pathology	8	11.3	31	43.7	12	16.9	20	28.2	**0.0001**
Soft tissue tumors	51	11.5	113	25.6	165	37.3	113	25.6	**3.48E−05**

**P** < 0.05 indicated a statistically significant difference and they are shown in bold

**Table 5:** Sex distribution among study categories

Categories	Sex distribution				*P* value
	Male		Female		
	N	%	N	%	
Allergies and immunologic disease	1	100.0	0	0.0	**0.3127**
Bone pathology	49	48.5	52	51.5	**0.8274**
Dermatologic diseases, immunologic	0	0.0	2	100.0	**0.1607**
Developmental, non-odontogenic cyst	6	54.5	5	45.5	**0.7390**
Epithelial Pathology	5	62.5	3	37.5	**0.4621**
Odontogenic cysts	170	55.6	136	44.4	**0.0135**
Odontogenic tumor Benign	56	57.7	41	42.3	**0.0915**
Periodontal diseases	9	40.9	13	59.1	**0.4130**
Periapical diseases	23	48.9	24	51.1	**0.9316**
Salivary gland pathology	23	32.4	48	67.6	**0.0028**
Soft tissue tumors	207	46.8	235	53.2	**0.1407**

**P** < 0.05 indicated a statistically significant difference and they are shown in bold

A statistically significant difference between the different lesion locations was described in all the reported lesions except for allergies and immunologic disease, dermatologic diseases, and periodontal diseases in Table 6.

**Table 6:** Location distribution among study categories

Categories	Location distribution								*P* value
	Lip	Buccal mucosa	Tongue	Palate	Mouth floor	Gingiva alv.ridge	man	max	
Allergies and immunologic disease	(0) 0.0%	(0) 0.0%	(0) 0.0%	(0) 0.0%	(0) 0.0%	(0) 0.0%	(1) 100.0%	(0) 0.0%	**0.915**
Bone pathology	(0) 0.0%	(0) 0.0%	(0) 0.0%	(1) 1.0%	(0) 0.0%	(2) 2.0%	(50) 49.5%	(48) 47.5%	**1.09E−17**
Dermatologic diseases, immunologic	(0) 0.0%	(2) 100.0%	(0) 0.0%	(0) 0.0%	(0) 0.0%	(0) 0.0%	(0) 0.0%	(0) 0.0%	**1.55E−09**
Developmental, non-odontogenic cyst	(1) 9.1%	(0) 0.0%	(0) 0.0%	(0) 0.0%	(2) 18.2%	(0) 0.0%	(1) 9.1%	(7) 63.6%	**5.49E−06**
Epithelial pathology	(2) 25.0%	(1) 12.5%	(3) 37.5%	(1) 12.5%	(0) 0.0%	(1) 12.5%	(0) 0.0%	(0) 0.0%	**6.32E−11**
Odontogenic cysts	(0) 0.0%	(0) 0.0%	(0) 0.0%	(0) 0.0%	(0) 0.0%	(2) 0.7%	(157) 51.3%	(147) 48.0%	**1.13E−81**
Odontogenic tumor Benign	(0) 0.0%	(0) 0.0%	(0) 0.0%	(0) 0.0%	(0) 0.0%	(3) 3.1%	(63) 64.9%	(61) 32.0%	**1.68E−19**
Periodontal diseases	(0) 0.0%	(0) 0.0%	(0) 0.0%	(0) 0.0%	(0) 0.0%	(22) 100.0%	(0) 0.0%	(0) 0.0%	**3.88E−08**
Periapical diseases	(0) 0.0%	(0) 0.0%	(0) 0.0%	(0) 0.0%	(0) 0.0%	(0) 0.0%	(24) 51.1%	(23) 48.9%	**4.61E−08**
Salivary gland pathology	(49) 69.0%	(4) 5.6%	(0) 0.0%	(7) 9.9%	(10) 14.1%	(0) 0.0%	(1) 1.4%	(0) 0.0%	**2.5E−117**
Soft tissue tumors	(29) 6.6%	(32) 7.2%	(20) 4.5%	(22) 5.0%	(3) 0.7%	(324) 73.3%	(6) 1.4%	(6) 1.4%	**1.8E−167**

**P** < 0.05 indicated a statistically significant difference and they are shown in bold

Concerning malignancy, a total of 19 lesions were diagnosed with the higher occurrence being reported in soft tissue (10 cases) followed by salivary gland (5 cases) and bone (4 cases) in Table 7.

**Table 7:** Distribution and demographic data of the malignant lesions in the study

Category	Diagnosis	Age				Sex		Location					N (%)
		0–5	6–10	11–15	16–18	Male	Female	Palate	Mouth floor	Gingiva alv ridge	man	max	
Malignant bone pathology	Osteosarcoma	0	1	1	1	2	1	0	0	0	0	3	3 (15.8%)
Malignant bone pathology	Chondrosarcoma	0	0	1	0	0	1	0	0	0	1	0	1 (5.3%)
Malignant salivary gland pathology	Mucoepidermoid carcinoma	0	3	0	1	4	0	4	0	0	0	0	4 (21.1%)
Malignant salivary gland pathology	Adenocarcinoma			1			1				1		1 (5.3%)
Malignant soft tissue tumors	Lymphoma	3	2	0	0	2	3	0	1	1	1	2	5 (26.3%)
Malignant soft tissue tumors	Plasmacytoma				1		1					1	1 (5.3%)
Malignant soft tissue tumors	Embryonal rhabdomyo sarcoma	1					1				1		1 (5.3%)
Malignant soft tissue tumors	Fibrosarcoma				1	1				1			1 (5.3%)
Malignant soft tissue tumors	Hemangio endothelioma				1		1			1			1 (5.3%)
Malignant soft tissue tumors	Alveolar soft tissue sarcoma		1			1				1			1 (5.3%)
Total malignant tumors		4	7	3	5	10	9	4	1	4	4	6	19 (100%)

## Discussion

The cumulative evidence from histopathological-related cross-sectional investigations on oral and maxillofacial lesions gives accurate data with negligible clinical diagnosis biases. These studies also emphasize the prevalence, incidence, and prognosis of a variety of diseases. Because local populations have diverse risk factors, georeferencing is especially important in this procedure [9].

For the first time, the present study offered a comprehensive evaluation of oral and maxillofacial pathology in an Egyptian pediatric population. Although this study only reveals the relative frequency of oral and maxillofacial lesions in this population, rather than the actual prevalence, it is valuable to both pathologists and pediatric dentists through determining the characteristics of these lesions in children and adolescents that provides the basis for proper diagnosis and treatment. Also, it can guide the differential diagnoses of pathology commonly seen in children [8, 10, 11].

Over 21 years, about 17.5% of all cases submitted for the histopathological examination were from children under the age of 18 years which was consistent with the following studies [10–13]. On the contrary, other studies reported that pediatric oral biopsies were less than 10% of all cases [8, 14–16]. This can be attributed to the difference in inclusion criteria, including age range or locations, population’s genetic background, geographical area, study period, and type of institution [17].

According to previous studies [6, 18, 19], four age groups were included in the present study to represent a true pediatric population. Also, a smaller pediatric age range was chosen in order to concentrate on the incidence of lesions observed in the newly-erupted permanent dentition and its associated growth and development of its supporting structures [19, 20].

About 37.2% of patients were found in the age group of 11–15 years and 30.2% in the age group of 16–18 years which were in agreement with the previous studies [6, 19, 21]. This finding can be explained by the fact that children and adolescents develop rapidly which affects the growth potential of neoplasms and cysts, as well as rendering this population more likely to acquire inflammatory lesions. Also, this phase is characterized by intense odontogenic activity, a fact that may be related to the occurrence of numerous lesions [15].

Another explanation is that older pediatric patients present a higher frequency of lesions, not only as a consequence of the epidemiological features of each disease but also because clinicians tend to postpone biopsies or surgeries considering that most of the pediatric lesions are reactive or benign in nature [16].

The present study showed an almost equal distribution between sexes similar to several studies [6, 11, 19, 22]. This fact may be attributed to the concern and care of the family about the oral health of children and adolescents, irrespective of sex [14].

The majority of the cases were found in the gingiva (31.9%), according to the site distribution analysis which was consistent with the following studies [17, 22]. On the contrary, several studies reported the lips as the most commonly affected site, representing 34.5% of the lesions [14, 15] and other studies reported the maxilla to be the most affected site for all types of oral and maxillofacial pathology [23, 24]. The variation in the distribution of oral and maxillofacial pathology by anatomical position can be due to the absence of standardization regarding the analysis of anatomical locations affected by oral and maxillofacial lesions [25].

According to the findings of this study, the prevalence of oral and maxillofacial lesions in children has increased over the years with almost half of the cases 549 (49.5%) were centered in the period from 2014 to 2019. This elevation in the number of pediatric biopsies can be related to genetic factors and changes in lifestyle. Also, this may suggest a higher awareness of pediatric oral health in our country [6].

There was no agreement on the classification of oral lesions into groups and subgroups in retrospective epidemiological studies of oral lesions from children's biopsies [15]. In the current study, obtained pathological diagnoses were grouped into 11 categories according to the classification proposed by World Health Organization [26] and Neville et al. [27].

Regarding the lesions categories, reactive soft tissue lesions were the most prevalent category in the present study (35.8%) which were in accordance with the following studies [6, 17, 24]. This finding may be attributed to their symptomatic nature compared to other lesions [17].

The majority of reactive lesions in the present study were in the 11–15 years age range similar to the previous studies [17, 19, 28]. Most of these lesions developed on the gingival mucosa, indicating the involvement of tooth pathology and status of dentition in the pathogenesis of at least some of them [29].

Moreover, pyogenic granuloma was the most frequently encountered lesion in this category followed by peripheral giant cell granuloma, and irritational fibroma. They also were ranked the 2nd, 3rd, and 4th most common lesions in the study population. These findings were consistent with the previous studies [24, 29, 30] and can be attributed to poor oral hygiene and calculus formation [17, 22].

On the contrary, the benign soft tissue lesions represented only (3.2%) of the lesions where hemangioma was the most common connective tissue benign tumor among the study sample and ranked the 18th in accordance with several studies [17, 22, 24].

This can be justified by the fact that many cases of hemangioma are clinically diagnosed, and not always be biopsied. Therefore, the occurrence of hemangioma might be even higher than the number of cases reported [24].

The odontogenic cysts including both inflammatory and developmental were the second and the third most prevalent categories in the present study representing 19.2% and 8.4% respectively. The prevalence of odontogenic cysts (27.6%) was higher in comparison to non-odontogenic cysts (1%) as reported in the majority of studies [11, 30, 31].

In the present study, the majority of cystic lesions were observed in the 11–15 years age range followed by the 16–18 years age range which was consistent with the previous studies [17, 31]. These lesions were commonly diagnosed in the mandible followed by the maxilla which was in agreement with the previous studies [17, 22].

The inflammatory radicular cyst was the most encountered lesion in the inflammatory odontogenic cyst category and ranked the 1st most common lesion in the study population which was in line with the following studies [8, 22, 30]. This finding may be explained by the high prevalence of dental caries and unsatisfactory oral health conditions, which unfortunately are still common in less developed countries [8, 15].

However, several studies reported a lower prevalence of radicular cyst [15]. This could be attributed to the fact that most radicular cyst cases are detected and treated clinically rather than being sent for a biopsy on a regular basis, particularly if the lesion is removed and disposed of after tooth extraction of the primary dentition [13, 15].

The reactive salivary gland pathology represented the fourth prevalent category among the study sample (5.5%) which was similar to several studies [6, 8]. It is considered the most frequent lesion of the salivary glands with a greater presence in children who are particularly vulnerable, especially in the lower lip area, mainly due to its etiological association with traumatic factors [8].

Mucous extravasation cyst (mucocele) accounted for the majority of reactive salivary gland pathologies and ranked the 5th most common lesions in the study sample which were in accordance with several studies [23, 29, 30]. The high incidence of mucus extravasation phenomenon could have resulted from trauma, such as lip or cheek bite, to the salivary glands [31].

Even though salivary tumors are generally uncommon in study populations (1%), the ratio of benign to malignant salivary tumors was 1:1. Therefore, any persistent swelling of unknown etiology within the salivary glands requires urgent investigation [29].

Benign epithelial odontogenic tumors represented the highest prevalence among odontogenic tumors (4.8%) where ameloblastoma was the most prevalent lesion in the benign odontogenic tumor category representing 2.8% of the study sample and ranked the 10th most common lesion among submitted samples which were in accordance with several studies [11, 15]. On the contrary, other studies reported that odontoma was the most common odontogenic tumor which ranked second to ameloblastoma in our study [8, 22].

This can be explained by the fact that most odontomas are asymptomatic lesions and the majority of people in developing countries do not undergo routine radiographic examination. Only when the lesion produces symptoms or disfigurement would patients seek medical attention [11].

Giant cell lesions (4.4%) and fibro-osseous (3.4%) bone lesions represented the major categories in bone pathology while benign bone pathology represented only 0.9% of the lesions. Central giant cell lesions and ossifying fibroma had the higher occurrence in their categories and ranked the 7th and 9th most common lesions. These findings were in accordant with the following studies [21, 30] and can be attributed to the fact that those lesions cause jaw expansion and this causes patients to seek medical attention [11].

Periapical lesions represented 47 (4.2%) of the total lesions where periapical granuloma presented as the most encountered lesion in its category and ranked the 6th most common lesion in the present study which was in line with the previous studies [10, 32]. This could be explained by the fact that the anatomy of primary teeth could help the faster progression of pulp pathology into the periapical area [33].

Oral lesions that conceal underlying systemic disorders like immunologic diseases were rare among pediatric oral pathology representing only 0.1% of lesions which was consistent with a previous study [29].

Despite the majority of lesions observed in this pediatric group are benign and require minor treatment, it is important to note that oral malignancies do occur in children at a rate equivalent to global trends. Malignant lesions represented 1.7% of total biopsies in the study population, where 26.3% of them were lymphoma, 21.1% were Mucoepidermoid carcinoma, and 15.8% were Osteosarcoma. These findings were consistent with several studies [8, 23, 29].

Other studies described a higher prevalence of malignant lesions in children, which was probably influenced by the endemic character of certain neoplasms such as Burkitt’s lymphoma [22, 29, 30].

The frequency of oral and maxillofacial pathological lesions among Egyptian children increased over the years but still showed a similar trend to that reported in previous studies. The majority of lesions detected were benign in nature with a very low number of malignant lesions. The majority of the lesions were in the category of reactive and inflammatory lesions with most occurring in the third age group (11–15 years).

In conclusion, the prevalence of oral and maxillofacial pathological abnormalities in Egyptian children grew with time but remained consistent with earlier researches. The vast majority of lesions discovered were benign, with only a few malignant tumors. Most of the lesions were reactive and inflammatory, with the majority of them appearing in the third age group (11–15 years).

## Data Availability

The datasets used during the current study are available from the corresponding author on reasonable request.
